# Prehospital Care Duration and 30‐Day Mortality Among Septic Shock

**DOI:** 10.1155/emmi/6071570

**Published:** 2026-04-01

**Authors:** Romain Jouffroy, Vincent Garrouste, Basile Gilbert, Stéphane Travers, Emmanuel Bloch-Laine, Patrick Ecollan, Vincent Bounes, Josiane Boularan, Benoit Vivien, Papa Gueye

**Affiliations:** ^1^ LI^2^RSO, Orléans University, Orléans University Hospital, Orléans, 45100, France; ^2^ Emergency Department, SAMU, Orléans La Source University Hospital, Orléans, France; ^3^ Center for Research in Epidemiology and Population Health, U1018 INSERM, Paris Saclay University, Gif-Sur-Yvette, France; ^4^ Institute for Biomedical Research and Sports Epidemiology, EA7329, INSEP, Paris University, Paris, France; ^5^ Paris Fire Brigade, French Military Health Service, Paris, France; ^6^ Department of Emergency Medicine, SAMU 31, University Hospital of Toulouse, Toulouse, France, chu-toulouse.fr; ^7^ Emergency Department, Cochin Hospital, Paris, France; ^8^ Emergency Department, SMUR, Hôtel Dieu Hospital, Paris, France; ^9^ Intensive Care Unit, SMUR, Pitie Salpêtriere Hospital, 47 Boulevard de L′Hôpital, 75013, Paris, France; ^10^ SAMU 31, Castres Hospital, Castres, France; ^11^ Intensive Care Unit, Anaesthesiology, SAMU, Necker Enfants Malades Hospital, Assistance Publique-Hôpitaux de Paris, Paris, France, aphp.fr; ^12^ SAMU 972 La Martinique Pierre Zobda University Hospital, Quitman Hospital, Fort-de-France, Martinique, France; ^13^ UR5_3 PC2E Cardiac Pathology, Environmental Toxicity, and Envenomation, Antilles University, Pointe-à-Pitre, Guadeloupe, France

**Keywords:** antibiotic, hemodynamic optimization, mortality, prehospital, septic shock

## Abstract

**Background:**

Septic shock may occur in the prehospital setting requiring a prehospital mobile intensive care unit (MICU) intervention. The prehospital MICU is equipped to deliver antibiotics and hemodynamic optimization on scene in order to implement effective treatment measures within the initial hour. The study objective is to assess the relationship between the duration of prehospital care and 30‐day mortality among patients with septic shock who received MICU prehospital antibiotic and norepinephrine administration.

**Methods:**

From 2016, May, to 2021, March, patients with septic shock managed by a prehospital MICU of nine French hospital centers were retrospectively analyzed. Multivariate logistic regression and propensity score analysis with the inverse probability weighting (IPTW) method were used to assess the association between prehospital care duration and 30‐day mortality.

**Results:**

Five‐hundred and thirty patients were analyzed among which 341 (64%) were males, and the mean age was 70 ± 15 years old. The 3 main presumed sepsis origins were pulmonary, digestive, and urinary for 43%, 25%, and 17% of the patients, respectively. One‐hundred and thirty‐two patients (25%) received prehospital antibiotic and 155 (29%) norepinephrine administration with a median dose of 1.0 [0.5–2.0] mg.h^−1^ within the first prehospital hour. The 30‐day mortality was 31%. The mean prehospital care duration was 71 ± 34 min. Multivariate logistic regression analysis revealed an association between prehospital care duration and 30‐day mortality rate: adjusted odds ratio (aOR) = 1.01 [1.00–1.02], *p* = 0.017. Multivariate logistic regression adjusted on the same potential confounders reported an association between prehospital care duration < 60 min and 30‐day mortality: aOR = 0.64 [0.41–0.99], *p* = 0.044. Log binomial regression weighted with the IPTW revealed an association between prehospital care duration < 60 min and 30‐day mortality: aOR = 0.56 [0.46–0.67], *p* < 10^−3^.

**Conclusion:**

The present study reports the association between prehospital care duration and 30‐day mortality among septic shock patients who received MICU prehospital antibiotic and norepinephrine administration.

**Key Findings**
 Prehospital care duration is positively associated with 30‐day mortality among septic shock patients cared for by a prehospital mobile intensive care unit. Antibiotic therapy and norepinephrine administration within the first hour are associated with 30‐day mortality decrease among this population.

## 1. Introduction

Every year, sepsis, a dysregulated response to infection, leads to millions of deaths [1, 2]. One‐third to one‐half of all in‐hospital deaths [3] are related to sepsis, with a mean 1‐month mortality rate close to 25% up to 50% for septic shock [4–6]. Consequently, international sepsis guidelines recommend, instead of a single treatment, a bundle of care including prompt identification, serum lactate measurement, peripheral blood cultures sampling, early antibiotic, and fluid resuscitation with ongoing hemodynamic support to reach a mean arterial pressure of at least 65 mmHg [7]; in other words, hemodynamic optimization. This bundle of care bundle improves sepsis‐related outcomes despite remaining debate [8, 9]. For the most severe sepsis form, i.e., septic shock, the “1‐h bundle” must be started within the first hour after sepsis recognition [1, 7, 10]. Among this bundle, early antibiotic and hemodynamic optimization [11–13] are the interventions mainly associated with improved sepsis outcomes [1, 9, 14–18].

Sepsis primarily manifests in the prehospital setting [19], with a median time of 1 hour preceding hospital admission [14]; thus, the question of the impact of prehospital antibiotic and hemodynamic optimization needs to be clarified. Despite different organizations worldwide, prehospital emergency medical services can deliver prehospital antibiotic and optimize the hemodynamic status [19].

In the current study, we assessed the relationship between the duration of prehospital care and 30‐day mortality among patients with septic shock who received mobile intensive care unit (MICU) prehospital antibiotic and norepinephrine administration.

## 2. Methods

### 2.1. Patients

As previously reported [20], in France, the prehospital emergencies management is based on the SAMU (Urgent Medical Aid Service), a public health control organization with a central dispatch center and prehospital MICU. Prehospital MICU, composed by a driver, a nurse, and an emergency physician equipped to support main organs deficiency [21], may be dispatch to the scene to provide medical assistance when necessary.

From 2016, May, to 2021, March, all septic shock patients suffering from septic shock according to the 2012 sepsis‐2 conference criteria [22] cared by a MICU of 9 French hospital centers (Necker‐Enfants malades Hospital, Lariboisière Hospital, La Pitié Salpêtrière Hospital, Hotel Dieu Hospital, APHP, Paris, France; Paris Fire Brigade, Paris, France; Toulouse University Health Centre, Toulouse, France, La Martinique University Health Centre, Fort de France, France, and the Castres Hospital, Castres, France) were retrospectively analyzed. Patients younger than 18 years, and/or pregnant, and/or with serious comorbid conditions with an unknown prehospital life support and/or with guardianship or curatorship were not included. As previously reported, we choose the operative sepsis‐2 definition considering a septic shock a condition of refractory hypotension despite vascular filling or normotension with hypoperfusion signs because prehospital lactatemia assessment was not available in all MICU [23].

As previously reported, patients’ demographic characteristics (age, weight, height, and gender), presumed prehospital origin of sepsis, initial prehospital (e.g., the first MICU contact), and final prehospital (e.g., at the end of prehospital stage) vital sign values (systolic (SAP), diastolic (DAP), and mean arterial pressure (MAP)) were measured with a noninvasive automated device in all centers. Heart rate (HR), pulse oximetry (SpO2), respiratory rate (RR), temperature and Glasgow coma scale (GCS), plasma blood glucose concentration, duration of prehospital care, and prehospital treatments delivered (antibiotic type and dose and fluid volume expansion type and dose, as well as catecholamine type and dose) were collected from MICU prehospital medical reports [22]. Major comorbidities, i.e., hypertension, coronary heart disease (CHD), chronic cardiac failure (CCF), chronic renal failure (CRF), chronic obstructive pulmonary disease (COPD), diabetes mellitus, and history of cancer were also collected to take into account the underlying condition. Length of stay (LOS) in the ICU, in‐hospital LOS, and 30‐day mortality were retrieved from medical reports in case of in‐hospital death or by call when the patient was discharged from the hospital. Sequential Organ Failure Assessment (SOFA) score and simplified acute physiology score (SAPS‐2) were calculated 24 h after ICU admission [24].

A standardized abstraction template established prior the study and a data check identifying no error were used to minimize data abstraction bias [25].

### 2.2. Ethical Considerations

The study was approved by the French Society of Anesthesia and Intensive Care ethics committee on December 12^th^, 2017 (Ref number: IRB 00010254–2017–026). According to the French law, for this noninterventional retrospective observational study, the ethical committee waived consent of patients.

### 2.3. Statistical Analysis

Results are expressed by mean with standard deviation for quantitative parameters with a Gaussian distribution, by median with interquartile range (Q1–Q3) for parameters with a nonnormal distribution and with absolute value and percentage for qualitative parameters. The primary outcome was the 30‐day mortality rate.

First, univariate analyses were performed to assess the relationship between each covariate and 30‐day mortality rate. Prehospital care duration was analyzed sequentially as a continuous variable, then as a categorical variable (4 quartiles: 1^st^, 2^nd^, and 3^rd^ with 4^th^ quartile as reference), and finally, as a binary variable using a 60‐min cutoff, all within similar analytical frameworks.

Second, a multivariate logistic regression analysis was performed to evaluate the relationship between 30‐day mortality rate and prehospital care duration (quantitative variable). Age, hypertension, CHD, CCF, CRF, COPD, diabetes mellitus and history of cancer, prehospital antibiotic, prehospital fluid volume expansion prehospital norepinephrine administration, prehospital duration, and center were considered as potential confounders. The selection of variables was predicated on the premise that they could influence the “30‐day mortality rate” (outcome) and/or the prehospital duration (explanatory factor) of the multivariate logistic regression.

Third, a multivariate logistic regression analysis was performed to evaluate the relationship between 30‐day mortality rate and prehospital care duration< 60 min using the same potential confounders covariates. The “first hour” cutoff was selected in accordance with the Surviving Sepsis Campaign guidelines [7].

Fourth, a log binomial regression model was performed after using the inverse probability of treatment weighting (IPTW) propensity score method, with the same variables considered as potential confounders.

Results were expressed as adjusted odds ratio (aOR) with (95 CI).

All tests were 2‐sided with a statistically significant *p* *value*  < 0 0.05.

All analyses were performed using R 3.4.2 (http://www.R‐project.org; the R Foundation for Statistical Computing, Vienna, Austria).

## 3. Results

### 3.1. Patient Characteristics

Five‐hundred and thirty patients were analyzed, among which 341 (64%) were males, and the mean age was 70 ± 15 years old between 2016, May, and 2021, December (Table 1).

**TABLE 1:** Population characteristics. Results are expressed by mean and standard deviation for quantitative parameters (normal distribution), by median and interquartile range for quantitative parameters (non‐gaussian distribution) and, by absolute value and percentage for qualitative parameters. P‐value corresponds to the comparison between deceased and alive patients on day‐30.

	Overall population (n = 530)	Alive (n = 366)	Deceased (n = 164)	**p** value
Age (years)	70 ± 15	68 ± 15	73 ± 14	**< 10** ^**−**^ **^3^**
Male gender	341 (64%)	243 (66%)	98 (60%)	0.141
Weight (kg)	74 ± 20	75 ± 20	70 ± 20	**0.014**
Height (cm)	170 ± 12	170 ± 12	169 ± 9	0.339

*Initial prehospital values*				
SBP (mmHg)	97 ± 30	99 ± 30	93 ± 30	0.055
DBP (mmHg)	58 ± 19	59 ± 19	55 ± 20	0.069
MAP (mmHg)	71 ± 22	72 ± 22	68 ± 22	0.064
HR (beats.min^−1^)	114 ± 28	115 ± 27	113 ± 31	0.463
RR (movements.min^−1^)	31 [22 – 36]	28 [22 – 35]	31 [25 –38]	**0.012**
Pulse oximetry (%)	91 [85 – 96]	93 [86 – 96]	90 [83 –95]	**0.005**
Body core temperature (°C)	38.3 [36.5 – 39.2]	38.4 [36.7 – 39.3]	38.1 [36.0 –39.0]	**0.018**
Glycemia (mmol.L^−1^)	8.8 [6.3 – 12.0]	8.9 [6.7 – 12.3]	7.9 [5.7 –11.2]	**0.014**
Glasgow coma scale	15 [12 – 15]	15 [13 – 15]	14 [11 – 15]	**0.002**
Blood lactate level (mmol.L^−1^)	5.8 ± 3.4	5.6 ± 3.2	6.3 ± 3.6	0.071
Prehospital fluid expansion (mL)	932 ± 573	944 ± 588	907 ± 542	0.523
Norepinephrine administration	155 (29%)	104 (28%)	51 (32%)	0.530
Norepinephrine dose (mg.h^−1^)	1.1 [0.5 – 2.0]	1.0 [0.6 – 2.0]	1.0 [1.0 – 2.0]	**0.041**
Prehospital AB administration	132 (25%)	97 (27%)	35 (21%)	0.205
Prehospital duration (min)	71 ± 34	69 ± 33	74 ± 35	0.111

*Prehospital suspected septic shock origin*				
Pulmonary	230 (43%)	144 (39%)	86 (52%)	**0.039**
Digestive	130 (25%)	88 (24%)	42 (26%)	0.989
Urinary	88 (17%)	70 (19%)	18 (11%)	0.989
Cutaneous	33 (6%)	26 (7%)	7 (4%)	0.989
Meningeal	11 (2%)	9 (2%)	2 (1%)	0.989
Gynaecological	3 (0.5%)	3 (1%)	0 (0%)	1.000
ENT	2 (0.5%)	1 (0.5%)	1 (1%)	0.988
Cardiac	2 (0.5)	2 (1%)	0 (0%)	1.000
Unknown	31 (6%)	23 (7%)	8 (5%)	0.989

*Final prehospital values*				
SBP (mmHg)	106 ± 25	109 ± 25	102 ± 27	0.056
DBP (mmHg)	62 ± 18	63 ± 18	60 ± 18	0.058
MAP (mmHg)	77 ± 19	78 ± 19	74 ± 20	0.064
HR (beats.min^−1^)	107 ± 25	107 ± 25	106 ± 28	0.396
RR (movements.min^−1^)	26 [19 – 30]	24 [17 – 30]	26 [20 – 34]	**0.012**
Pulse oximetry (%)	97 [94 – 99]	97 [95 – 99]	97 [93 – 98]	**0.003**
Body core temperature (°C)	38.1 [36.0 – 39.0]	38.0 [37.0 – 39.0]	37.0 [35.0 – 38.0]	**0.008**
Glycemia (mmol.L^−1^)	8.1 [6.1 – 10.1]	8.0 [6.1 – 10.0]	8.0 [6.1 – 10.6]	0.470
Glasgow coma scale	15 [14 – 15]	15 [14 – 15]	14 [12 – 15]	**< 10** ^**−**^ **^3^**
Blood lactate level (mmol.L^−1^)	4.2 ± 3.3	3.5 ± 2.9	5.7 ± 3.8	**< 10** ^**−**^ **^3^**
In‐ICU length of stay (days)	4 [2 – 8]	4 [2 – 9]	3 [1 – 7]	**0.007**
In‐hospital length of stay (days)	10 [5–18]	13 [7 – 21]	5 [2 – 11]	**< 10** ^**−**^ **^3^**
SOFA score	6 [3 – 9]	5 [3 – 8]	7 [4 – 10]	**< 10** ^**−**^ **^3^**
SAPS2 score	61 ± 22	54 ± 18	71 ± 21	**< 10** ^**−**^ **^3^**

*Comorbidities*				
Hypertension	230 (43%)	159 (44%)	71 (43%)	0.974
Coronary heart disease	104 (20%)	64 (18%)	40 (24%)	0.065
Chronic cardiac failure	134 (25%)	74 (20%)	60 (37%)	**< 10** ^**−**^ **^3^**
Chronic renal failure	151 (29%)	109 (30%)	42 (26%)	0.069
COPD	75 (14%)	45 (12%)	30 (18%)	0.144
Diabetes mellitus	151 (28%)	81 (22%)	70 (43%)	0.326
Cancer history	186 (35%)	156 (43%)	30 (18%)	**0.015**

*Note:* The *p* value in bold indicates a *p* value of less than 0.05 between living and deceased patients on Day 30.

Abbreviations: ABT = antibiotic therapy, COPD = chronic obstructive pulmonary disease, DBP = diastolic blood pressure, ENT = ear nose throat, HR = heart rate, ICU = intensive care unit, MBP = mean blood pressure, min = minutes, RR = respiratory rate, SAPS2 = simplified acute physiology score 2nd version, SBP = systolic blood pressure, SOFA= sequential organ failure assessment.

Suspected prehospital septic shock were mainly pulmonary, digestive, and urinary: respectively, 43%, 25%, and 17%. For 31 patients (6%), the prehospital septic shock origin was unknown (Table 2).

**TABLE 2:** Suspected prehospital septic shock origins.

Origin	*n* (percentage)
Pulmonary	230 (43%)
Digestive	130 (25%)
Urinary	88 (17%)
Cutaneous	33 (6%)
Meningeal	11 (2%)
Gynaecological	3 (0.5%)
ENT	2 (0.5%)
Cardiac	2 (0.5)
Unknown	31 (6%)

Abbreviation: ENT: ear nose throat.

One‐hundred and thirty‐two patients (25%) received prehospital antibiotic: 97 patients (27%) vs. 35 patients (21%) (*p* = 0.205) among alive and deceased patients on Day 30 without serious adverse event related to prehospital antibiotic administration. Among these patients, 98 patients (74%) received a 3^rd^ generation cephalosporin: 39% cefotaxime and 61% ceftriaxone.

Fluid expansion was based on crystalloids infusion for all patients with no significant difference between living and deceased patients: 945 ± 593 mL vs 907 ± 542 mL, respectively (*p* = 0.511; Table 1). One‐hundred and fifty‐five patients (29%) received norepinephrine administration, 104 (28%) vs. 51 (32%), *p* = 0.530, among alive and deceased patients on Day 30 with a median dose of 1.0 [0.5–2.0] mg h^−1^ within the first prehospital hour: 1.0 [0.6–2.0] vs 1.0 [1.0–2.0], *p* = 0.041, among alive and deceased patients on Day 30 (Table 1).

The 30‐day mortality was 31%.

The median ICU length of stay was 4 [2–8] days and the median length of stay in a hospital was 10 [5–18] days.

### 3.2. Main Measurement

The mean prehospital care duration was 71 ± 34 min without difference in the duration of prehospital stage between survivors and deceased patients (69 ± 33 min vs. 74 ± 35 min, respectively, *p* = 0.111; Table 1).

A total of 138 patients were managed within the first quartile [0–45] minutes, 124 within the second quartile [46–68] minutes, 132 within the third quartile [69–89] minutes, and 136 within the fourth quartile [90–205] minutes.

### 3.3. Multivariate Analyses

#### 3.3.1. Prehospital Care Duration (Quantitative Variable)

Multivariate logistic regression analysis adjusted on age, hypertension, CHD, CCF, CRF, COPD, diabetes mellitus and history of cancer, prehospital antibiotic, prehospital fluid volume expansion prehospital norepinephrine administration, prehospital duration, and center reported a significant association between prehospital care duration (quantitative variable) and 30‐day mortality: aOR = 1.01 [1.00–1.02], *p* = 0.017.

#### 3.3.2. Prehospital Care Duration (Categorical Variable)

Multivariate logistic regression analysis adjusted on the same previous potential confounders reported a significant association between 30‐day mortality and prehospital care duration quartiles (reference 1^st^ quartile, i.e., 0–45 min): 45 – 67 min: aOR = 2.43 [1.05–5.90], *p* = 0.043 (*n* = 124 patients), 68 – 89 min: aOR = 3.16 [1.47–7.33], *p* = 0.005 (*n* = 132 patients), and 90 – 205 min: aOR = 3.48 [1.55–8.29], *p* = 0.003 (*n* = 136 patients).

#### 3.3.3. Prehospital Care duration< 60 min

Multivariate logistic regression analysis adjusted on the same previous potential confounders reported a significant association between prehospital care duration < 60 min and 30‐day mortality: aOR = 0.64 [0.41–0.99], *p* = 0.044.

In addition, a significant 30‐day mortality increase was observed in patients with prehospital antibiotic and norepinephrine administration after the first hour: aOR = 1.57 [1.01–2.44], *p* = 0.044.

#### 3.3.4. Propensity IPTW Analyses

The log binomial regression weighted with the IPTW reported a significant association between prehospital care duration< 60 min and 30‐day mortality: aOR = 0.56 [0.46–0.67], *p* < 10^−3^.

## 4. Discussion

In this study, we observed a significant 30‐day mortality decrease among septic shock patients cared for by a prehospital MICU benefiting from a prehospital antibiotic and norepinephrine within the first hour.

The international guidelines for sepsis care emphasize the need for early recognition, severity assessment, and early treatment to improve sepsis outcome [1, 7, 10]. In this regard, evidence‐based medicine and clinical guidelines have also shown that, rather than a single treatment, a bundle of care is more efficient [1, 7, 10]. Among the bundle of care, 2 treatments have a consequent impact on sepsis outcome [1, 9, 14–18], especially for the critically ill patients, those with septic shock [14]: early antibiotic administration and early hemodynamic optimization [11–13]. Still, for the critically ill patients, it is recommended to start treatments within the first hour after diagnosis; in other words, to initiate “1‐h bundle” [1, 7, 10] when the treatment effect is the greatest [10, 26].

Given that approximately 70% of the sepsis cases occur in the prehospital setting, with a median time to hospital arrival of approximately 1 h [14], the MICU intervention enables the opportunity to initiate treatment at this stage. In France, the prehospital EMS based on SAMU with prehospital MICU allows to deliver care, antibiotic, and hemodynamic optimization early since the first hour [21].

Hemodynamic optimization aims to reach a mean arterial pressure of at least 65 mmHg [10, 26] with fluid volume expansion and norepinephrine infusion [1, 13]. A previous study reported that the earlier is the hemodynamic optimization, the better the outcome is [27].

More than antibiotics alone, sepsis treatment also relies on source control, which is not accessible in the prehospital setting and has a predominant effect on outcomes, for example, surgery for peritonitis [28]. The herein results are in line with previous ones observed in the hospital for early antibiotic positive on sepsis outcome. According to the literature, the administration of antibiotics in the early stages of treatment is associated with a decreased mortality rate and a reduced length of stay in the hospital [27, 29].

This study suffers from some limitations. This is a retrospective study precluding any conclusion for the reasons of prehospital antibiotic infusion and/or norepinephrine use. Data collection from prehospital and in‐hospital medical reports does not exclude the possibility of a misclassification bias. The French prehospital EMS specificity based on SAMU and MICU may affect the external validity. Intrinsically, the antibiotic spectrum (third generation cephalosporin) effective on most of the bacteria from pulmonary, digestive, and urinary community‐acquired agents may affect our study results. Finally, the statistical analysis performed does not allow any causal conclusion between outcome and covariates.

Beyond the study limits, it appears that for septic shock, a rapid severity assessment and the early initiation of treatment, i.e., since the prehospital phase, which may be prolonged beyond 1 h, is beneficial for the patient’s outcome.

## 5. Conclusion

In this study, we observed the association between prehospital care duration and 30‐day mortality among septic shock patients who received MICU prehospital antibiotic and norepinephrine administration. These preliminary results must be confirmed by larger prospective multicentric studies.

## Author Contributions

R.J., P.G., and B.V. conceived the study, prepared the initial protocol, analyzed the data, and drafted the manuscript.

R.J., V.G., E.B‐L., P.E., P.G., J.B., S.T., V.B., and B.G. collected the data.

## Funding

This research did not receive any specific grant from funding agencies in the public, commercial, or not‐for‐profit sectors.

## Disclosure

All authors read and approved the final version of the manuscript.

## Ethics Statement

The study was approved by the French Society of Anesthesia and Intensive Care ethics committee on 2017, December 12^th^ (Ref number: IRB 00010254–2017‐026).

## Consent

The authors have nothing to report.

## Conflicts of Interest

The authors declare no conflicts of interest.

## Data Availability

Data will be made available on request from the corresponding author.
